# Surgical Site Infections in Glioblastoma Patients—A Retrospective Analysis

**DOI:** 10.3390/jpm13071117

**Published:** 2023-07-10

**Authors:** Maximilian Scheer, Kai Spindler, Christian Strauss, Stefan Schob, Christian T. Dietzel, Sandra Leisz, Julian Prell, Stefan Rampp

**Affiliations:** 1 Department of Neurosurgery, University Hospital Halle, Ernst-Grube-Straße 40, 06120 Halle (Saale), Germany; 2 Department of Radiology, University Hospital Halle, Ernst-Grube-Straße 40, 06120 Halle (Saale), Germany; 3 Department of Radiation Oncology, University Hospital Halle (Saale), Ernst-Grube-Str. 40, 06120 Halle (Saale), Germany; 4 Department of Neurosurgery, Department of Neuroradiology, University Hospital Erlangen, Schwabachanlage 6, 91054 Erlangen, Germany

**Keywords:** surgical site infection, glioblastoma, irradiation, radicality, retrospective analysis, influencing factor

## Abstract

Surgical site infections (SSIs) after craniotomy lead to additional morbidity and mortality for patients, which are related to higher costs for the healthcare system. Furthermore, SSIs are associated with a longer hospital stay for the patient, which is particularly detrimental in glioblastoma patients due to their limited life expectancy. Risk factors for SSIs have already been described for craniotomies in general. However, there is limited data available for glioblastoma patients. As postoperative radiation influences wound healing, very early radiation is suspected to be a risk factor for SSI. Nevertheless, there are no data on the optimal timing of radiotherapy. To define risk factors for these patients, we analyzed our collective. We performed a retrospective analysis of all operations with histological evidence of a glioblastoma between 2012 and 2021. Open biopsy and tumor removal (gross total resection, subtotal resection) were included. Stereotactic biopsies were excluded. Demographic data such as age and gender, as well as duration of surgery, diameter of the trepanation, postoperative radiation with interval, postoperative chemotherapy, highest blood glucose level, previous surgery, ASA score, foreign material introduced, subgaleal suction drainage, ventricle opening and length of hospital stay, were recorded. The need for surgical revision due to infection was registered as an SSI. A total of 177 patients were included, of which 14 patients (7.9%) suffered an SSI. These occurred after a median of 45 days. The group with SSIs tended to include more men (57.1%, *p* = 0.163) and more pre-operated patients (50%, *p* = 0.125). In addition, foreign material and subgaleal suction drains had been implanted more frequently and the ventricles had been opened more frequently, without reaching statistical significance. Surprisingly, significantly more patients without SSIs had been irradiated (80.3%, *p* = 0.03). The results enable a better risk assessment of SSIs in glioblastoma patients. Patients with previous surgery, introduced foreign material, subgaleal suction drain and opening of the ventricle may have a slightly higher for SSIs. However, because none of these factors were significant, we should not call them risk factors. A less radical approach to surgery potentially involving these factors is not justified. The postulated negative role of irradiation was not confirmed, hence a rapid chemoradiation should be induced to achieve the best possible oncologic outcome.

## 1. Introduction

Craniotomies are occasionally followed by surgical site infections (SSIs), which are associated with increased morbidity and mortality. In addition to a prolonged hospital stay, there are also higher costs for the healthcare system [1]. Especially in glioblastoma patients with limited life expectancy, a prolonged hospital stay must be avoided. In addition, wound infections have been associated with reduced survival in glioblastoma patients [2]. Glioblastomas represent the most common and aggressive form of astrocytic brain tumors in adults [3]. Surgical treatment is an important pillar of therapy. Due to the invasive growth of this tumor, curative treatment is not possible. Despite multimodal therapy including radiation, chemotherapy and therapy with alternating electric fields, life expectancy remains poor and recurrences almost always occur [4,5,6]. Another operation is often considered even in the recurrence situation [7]. In the case of SSIs, the bone flap is usually removed. This often means a new operation in the interval and additionally a delay of the adjuvant therapy for the patient [8].

Previous studies on SSIs have included all craniotomies. Studies focusing on patients with glioblastoma and their risks are scarce. It is only known that an eloquent tumor location and adjuvant therapy generally promote complications and that a low Karnofsky performance score (KPS) and steroid administration favor SSIs [9,10].

Risk factors for the development of surgical site infections after craniotomy in general have been studied frequently. The infection rate after craniotomy has been reported in the literature to range from 2.47% to 15.3% and occurs on average after 40–50 days [11,12]. Furthermore, emergency procedures also showed a higher rate of SSIs [13]. Previous surgery, surgery duration of more than 4 h, cerebrospinal fluid (CSF) fistula, insertion of foreign material and an ASA score of more than two were identified as risk factors [1,11,12]. The presence of a malignant tumor was also associated with a higher rate of wound infections after craniotomy [12]. On the other hand, the introduction of perioperative antibiotic prophylaxis significantly reduced the infection rate after craniotomies [14].

Given the known risk factors for SSIs after craniotomies, the natural question is whether surgery for glioblastoma results in an increased risk of SSIs due to the opening of the ventricle or the introduction of foreign material. Other factors, such as the diameter of the trepanation, have not been studied. In addition, the correlation between SSIs, chemotherapy and irradiation has not been sufficiently investigated so far [1,11,12]. There is no doubt that irradiation affects wound healing through DNA damage and decreased angiogenesis, which can lead a wound healing disorder and to SSIs [15]. However, as for the timing of radiotherapy after surgery, there is little data in the literature, and the optimal timing for irradiation after surgery remains unclear. We studied our glioblastoma patient population who were treated between 2012 and 2021 in order to try to identify risk factors concerning SSIs.

## 2. Methods

### 2.1. Population

We retrospectively reviewed the records of all patients with histological evidence of a glioblastoma according to the WHO classification at the time of diagnosis [16,17,18], who had undergone surgery between 2012 and 2021. Perioperative antibiotic prophylaxis was regularly administered during all operations. The drug of choice was cefazolin, provided there were no allergies. During surgery, the scalp was shaved in the area of the planned trepanation and then disinfected for 10 min. The surgical area was covered with sterile drapes, and a foil was applied in the area of the skin incision. The bone flaps were fixed with a mini-plate screw system in all cases.

Inclusion criteria were a craniotomy with subsequent open or extended biopsy, subtotal or gross total resection and age over 18 years. Patients who received stereotactic biopsy were excluded. SSI was defined in accordance with the Centers for Disease Control and Prevention (CDC) criteria, as follows: purulent drainage from a surgical incision; organism identification by culture from fluid and evidence of abscess on images or surgical revision. The need for surgical revision due to infection was registered as an SSI and its interval was recorded. The bone flap was removed in case of infection. In the interval, the defect was covered with synthetic bone graft.

The study was reviewed and approved by the responsible ethics committee. As it is a retrospective analysis with anonymized data, no written consent was required.

### 2.2. Data Collection

**Figure 1:**
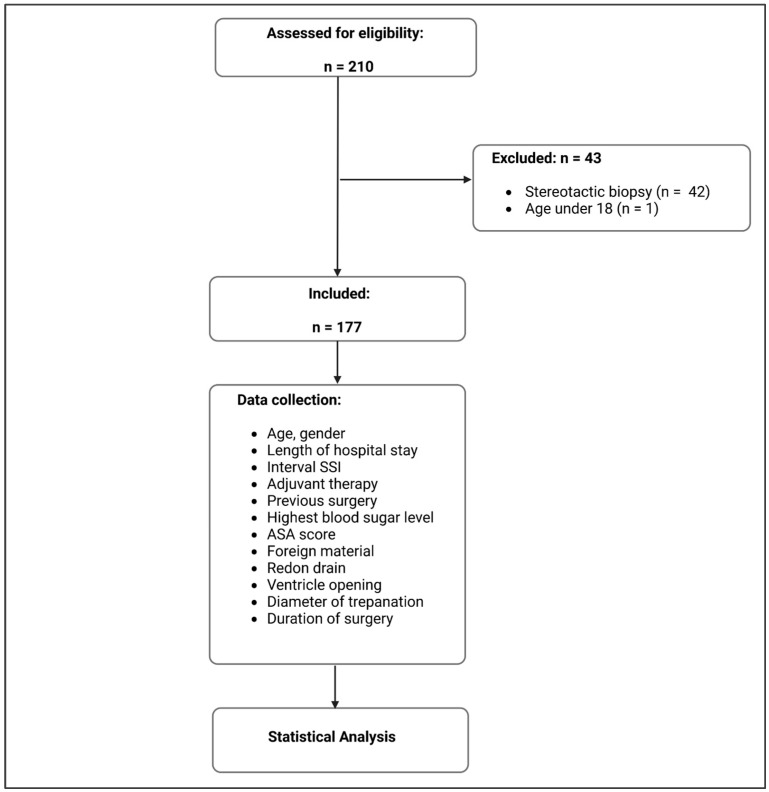
Flow chart on the progress of the study and the data analysis (created with BioRender^®^, Toronto, Canada).

Demographic data such as the age and gender of the patients as well as selected medical data were collected from the medical record. Operative data such as the duration of surgery, the largest diameter of the trepanation in the axial slice in a postoperative image and whether a subgaleal suction drain was implanted or the ventricle was opened during the surgery were also recorded. The use of artificial materials (bovine duraplastics or TachoSil^®^) was evaluated as foreign material. The bone fixation system was not considered foreign material. Adjuvant therapy such as chemotherapy and irradiation with its interval were also registered. In addition, other potential influencing factors such as the highest blood glucose level during the inpatient stay, previous operations, the preoperative American Society of Anesthesiologists (ASA) score [19] and the length of the hospital stay were recorded (Figure 1).

### 2.3. Statistical Analysis

Characteristics of groups with and without SSIs were compared using Fisher’s exact test and Student’s t-test. Statistical evaluation was conducted using R version 4.2.3 (R Core Team (2023). R: A language and environment for statistical computing. R Foundation for Statistical Computing, Vienna, Austria. URL https://www.R-project.org/; accessed on 7 April 2023).

## 3. Results

In total, data from 210 patients were reviewed for eligibility. Forty-three patients had to be excluded due to a stereotactic biopsy (n = 42) or age below 18 years (n = 1). Thus, data from a total of 177 patients could be evaluated, whose demographic and medical data are summarized in Table 1.

SSIs were recorded in a total of 14 (7.9%) patients. Accordingly, no SSI was detected in 163 patients (92.1%). Revision due to SSI was required after an average of 45 days (±41). Demographic data showed a gender distribution of 42.9% women (6) in the SSI group. In the Non-SSI group, the ratio was reversed with a proportion of 62.6% women (102), without this being significant (*p* = 0.163). The median age was comparable in both groups and was 59 years (±11) in the SSI group and 63 years (±11) in the Non-SSI group (*p* = 0.180).

The median length of hospital stay differed only marginally and was 18 days (±9) in the SSI group and 21 days (±10) in the Non-SSI group (*p* = 0.215). The highest blood glucose level during hospitalization, which was recorded as a parameter for derailed diabetes mellitus, was 13 mmol/L (±4) compared to 13 mmol/L (±5) in the Non-SSI group (reference 3.8–6.1 mmol/L) (*p* = 0.938). In both groups, the mean preoperative ASA score, as a measure of the patients’ physical condition, was identical with a value of 2 (*p* = 0.930).

The rate of prior surgery was almost double in the SSI group, with 7 (50.0%) compared to 46 (28.2%) in the Non-SSI group but was not significant (*p* = 0.125). Most patients received adjuvant chemotherapy: 10 patients (71.4%) in the SSI group and 105 patients (64.4%) in the Non-SSI group (*p* = 0.775).

The rate of postoperative irradiation was the only significant parameter in our analysis. This was performed in only 8 patients (57.1%) in the SSI group versus 131 patients (80.4%) in the Non-SSI group (*p* = 0.034). However, the interval to radiotherapy was almost identical in both groups, with a value of 22 days (±14) in the SSI group and 23 days (±9) in the Non-SSI group (*p* = 0.950). A majority of patients (73.5%—130/177 patients) were treated according to the classical Stupp protocol [6], i.e., a dose of 60 Gy cumulatively. The dose ranged from 36 Gy to 60 Gy and averaged 56.8 Gy.

Regarding surgical details, there was a trend towards more frequent implantation of foreign material in the SSI group, but without reaching statistical significance: 10 patients (71.4%) in the SSI group compared to 95 patients (58.3%) in the Non-SSI group (*p* = 0.405). A similar trend was seen regarding the implantation of a subgaleal suction drain: 6 patients (42.9%) in the SSI group and 59 patients (36.2%) in the Non-SSI group. This parameter was also not significant (*p* = 0.773). Similarly, the rate of intraoperative opening of a ventricle was not significant. This was documented in 6 patients (42.9%) in the SSI group and in 54 patients (33.1%) in the Non-SSI group (*p* = 0.558).

**Figure 2:**
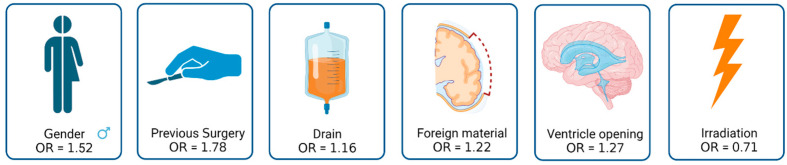
Factors with the greatest influence on SSIs measured using odds ratio (OR) (created with BioRender^®^).

The size of the trepanation based on the largest axial diameter in a postoperative image averaged 65 mm (±17) in the SSI group versus 64 mm (±18) in the Non-SSI group (*p* = 0.731). The mean duration of surgery was approximately the same in both groups and was 267 min (±120) in the SSI group versus 258 min (±87) in the Non-SSI group (*p* = 0.793). The details of all patients with SSIs are listed in Table 2. Factors with the greatest influence on SSIs in terms of odds ratio are summarized in Figure 2. Overall, the effects were rather small. For the male sex, the OR was 1.52, and prior surgery was associated with an OR of 1.78. We saw an OR of 1.16 for the insertion of a subgaleal drain. Insertion of foreign material or opening of a ventricle showed similar OR values with 1.22 and 1.27, respectively. Postoperative irradiation showed an OR value of 0.71 regarding SSIs.

As a side aspect, we have been able to observe a decrease in surgical cases due to the Corona pandemic in 2020 and 2021. Since 2017, our center has been certified as a neuro-oncology center. Since the year 2012, we have observed a continuous increase in surgical cases (glioblastomas) from an initial 21 to 35 cases per year in 2019. Then, in 2020 and 2021, there were only 23 and 22 cases, respectively.

## 4. Discussion

Risk factors after craniotomies in general with regard to SSIs have already been well studied. However, specific evidence for glioblastoma patients is sparse. This is however particularly relevant for these patients, as an SSI requires an interruption of therapy and shortens survival [20]. In most cases, removal of the autologous bone flap is required, as well as further surgery to cover the defect in the interval. This of course means a renewed risk of perioperative complications.

One measure that significantly reduced the rate of SSIs in all craniotomies, including glioblastoma patients, was perioperative antibiotic prophylaxis [14] as also applied in our study in all cases. Other methods such as intraoperative use of 5-ALA and cortical mapping also favor higher rates of gross total resection with positive impact on survival [2]. Other measures that contribute to low rates of SSIs include using antistaphylococcal skin antiseptic preoperatively, use of clippers rather than razors and intraoperative maintenance of normothermia and normoglycemia [21].

Regarding SSIs in glioblastoma, we found only two studies, one of which recorded only general complications. This was the case for older age and eloquent location [9]. In terms of age, our two groups were approximately equal. Based on our data, age does not seem to play a major role here. In the second study, lower preoperative KPS was independently associated with an elevated risk of postoperative SSI [10]. Due to the retrospective analysis, it was not possible to collect reliable KPS in this study. A possible expression of a reduced KPS, however, is the lower rate of postoperative irradiation in the SSI group in our cohort. The rate of postoperative irradiation was the only significant parameter in our analysis, with significantly more patients receiving irradiation in the group without SSI. This fact might be influenced since some patients experienced early onset of SSI about two weeks after surgery and therefore irradiation was not performed. Irradiation may impair wound healing through DNA damage, decreased angiogenesis, ischemia and abnormal collagen deposition [15] and thus early irradiation may interfere with wound healing, potentially promoting SSIs. However, there are no valid data in the literature on the optimal timing of irradiation. In animal models, irradiation was safe after 1 week, although this interval is likely to be significantly longer in humans [22]. In contrast, our data do not seem to show any negative influence of irradiation. The interval was the same in both groups, about 3 weeks from surgery to irradiation. Therefore, we conclude that this seems to be a safe time window. A majority of the patient collective (73.5%) was treated with 60 Gy according to the Stupp protocol. The mean applied postoperative radiation dose was 56.8 Gy, close to the Stupp protocol. From our data, it cannot be deduced whether the dose has an impact on SSIs.

Adjuvant chemotherapy was also shown to be an independent risk factor after craniotomy [23]. The rate of postoperative chemotherapy was relatively homogeneous in our cohort with nearly 2/3 of all patients receiving adjuvant chemotherapy. This factor did not seem to play a supporting role in SSIs.

Furthermore, McCutcheon et al. were able to show that the length of hospital stay over 30 days was a risk factor for SSIs after craniotomies [23]. This factor was almost identical in our two groups and was not significant in the analysis. In addition, diabetes mellitus is also considered an individual risk factor [24,25]. In our analysis, we used the highest blood glucose value during the inpatient stay as a benchmark for derailed diabetes mellitus. The values here were also very homogeneous, though relatively high and did not show differences between the groups.

The factor of trepanation diameter had not been considered in the literature so far. This parameter also differed only marginally in both groups and does not seem to play a relevant role for SSIs. What may be even more relevant than the size of the trepanation is the size of the access as well as the type of incision. For example, a reduction of SSIs in spine surgery was shown by introducing minimally invasive procedures. For our collective, due to the retrospective nature, the size of the access could not be accurately determined, so the size of the trepanation was used to be able to evaluate an objectifiable factor [26,27]

A review showed that revision due to SSIs was necessary in neurosurgical patients with a range from 8 to 854 days with a median of 42 days [12] An analysis by Grundy et al. also showed a comparable interval to the onset of SSI at 49 days [28]. Thus, the interval in our collective confirms the data in the literature on SSIs in craniotomy patients. The rate of SSIs after craniotomy is reported to be between 2% and 15% [10,24,29], i.e., the percentage of 7.9% observed in our study is well within this range. Male gender was also shown to be an independent risk factor in some studies [1,24]. In our population, there was a trend towards more men in the SSI group, but this was not significant. Other known risk factors for SSIs after craniotomy such as ASA score above 2, previous surgery, drain and foreign material were studied [1,11,12]. The ASA score was identical in both groups in our study with a value of 2, although GBM patients are often classified with a score > 3 [30]. The factors of previous surgery, Redon drain, ventricle opening and foreign material each showed a trend with increasing frequency in the SSI group, but again no significance was seen. A study using machine learning was able to show that CSF leakage and subcutaneous collection highly correlate with SSIs. However, it is unclear how these two factors are related to ventricular opening [31]. In our collective, ventricular opening had a slightly increased risk (OR 1.27). However, this effect was not significant. A statement on CSF leakage and subcutaneous collection is not possible due to the study design.

As a side aspect, we could see a decrease in surgical cases with glioblastoma patients in 2020 and 2021 due to the Corona pandemic. As Rucisnka et al. have pointed out, the pandemic resulted in postponed screening, diagnosis and treatment in many cancer patients. This fact is of course worrisome, as it must be assumed that many glioblastoma patients may not have received any therapy at all [32].

Some studies have also shown that tumor surgery increases the risk of SSIs [1,11,12]. Of course, the question arises whether the exact histology has an influence. It is difficult to say whether tumor entity also has an effect on SSI. All studies only differentiate according to the reason for the craniotomy (e.g., trauma or tumor). Histology is not considered further there [1,11,24]. We found only one study on childhood tumors where medulloblastoma was associated with an increased risk of SSIs [33].

To sum up, we conclude that the radicality of the surgery and the associated oncological benefit should be maintained. The study showed no results that argue against ventricular opening, implantation of a drain or foreign material.

This study has limitations since it is a retrospective analysis, which is partly associated with issues of data documentation. Factors such as diabetes mellitus or KPS were poorly documented, especially in the first years of the evaluation, and could not be taken into account. In addition, the absolute number of SSIs is small, so statements regarding risk factors are naturally limited. Even a few patients can influence a factor in one direction or another, which would possibly result in a different statement. Therefore, in our view, larger and prospective studies are useful to validate the factors mentioned.

## 5. Conclusions

**Figure 3:**
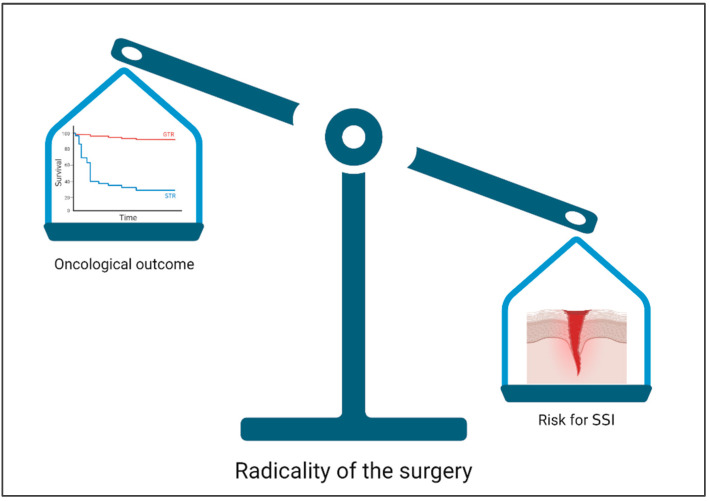
Possible influence of the radicality of the surgery with regard to oncological outcome and SSIs (created with Biorender^®^, Toronto, Canada).

In our analysis, we found that the factors of male gender, previous surgery, subgaleal drainage, foreign material and ventricular opening were more frequently present in the group with SSI and may therefore be classified as influencing factors. However, none of the listed factors showed a significant difference to the Non-SSI group and should not be classified as risk factors. Radicality of surgery appears to have little effect with respect to the risk of SSIs. We conclude that the oncologic benefit of radical surgery probably outweighs the risk of SSIs (Figure 3). However, the relatively small number of cases is limiting in our study. A negative impact of postoperative irradiation was not evident. A time window of 3 weeks after surgery seems to be safe and was associated with less SSIs in our population.

## Figures and Tables

**Table 1:** Baseline data.

	SSIs	No SSI	*p* Value	Test
Number	14 (7.9%)	163 (92.1%)		
Female	6 (42.9%)	102 (62.6%)	0.163	Fisher
Age (years, mean, SD)	59 (±11)	63 (±11)	0.180	*t*-Test
Hospital stay (days, mean, SD)	18 (±9)	21 (±10)	0.215	*t*-Test
Interval SSI (days, mean, SD)	45 (±41)			
Previous surgery	7 (50.0%)	46 (28.2%)	0.125	Fisher
Chemotherapy	10 (71.4%)	105 (64.4%)	0.775	Fisher
Irradiation	8 (57.1%)	131 (80.4%)	0.034	Fisher
Interval irradiation (days, mean, SD)	22 (±14)	23 (±9)	0.950	*t*-Test
Highest blood glucose level (mmol/L, mean, SD)	13 (±4)	13 (±5)	0.938	*t*-Test
ASA score (mean, SD)	2 (0–2)	2 (0–2)	0.930	Fisher
Foreign material	10 (71.4%)	95 (58.3%)	0.405	Fisher
Subgaleal suction drain	6 (42.9%)	59 (36.2%)	0.773	Fisher
Ventricle opening	6 (42.9%)	54 (33.1%)	0.558	Fisher
Diameter of trepanation (mm, mean, SD)	65 (±17)	64 (±18)	0.731	*t*-Test
Duration of surgery (min, mean, SD)	267 (±120)	258 (±87)	0.793	*t*-Test

**Table 2:** Listing of all patients with SSIs.

Number	Sex	Age (Years)	Stay (Days)	Interval SSI (Days)	Previous Surgery	Chemotherapy	Irradiation	Interval Irradiation (Days)	Highest Blood Glucose Level	ASA Score
1	female	56	32	22	no	no	no		19.5	2
2	female	52	33	65	yes	yes	yes	26	12.1	2
3	female	65	18	25	no	no	yes	23	12.0	3
4	female	76	35	16	no	no	no		18.9	3
5	male	55	17	44	no	yes	yes	13	17.0	2
6	male	72	17	21	no	yes	yes	13	16.1	3
7	male	43	11	172	no	yes	yes	20	16.0	2
8	female	48	12	68	yes	yes	no		13.0	2
9	female	50	10	63	yes	yes	yes	26	6.3	2
10	male	66	24	15	no	yes	yes	33	11.5	3
11	male	56	11	15	yes	yes	no		8.2	2
12	male	71	10	35	yes	no	no		10.5	3
13	male	71	10	24	yes	yes	no		14.0	2
14	male	47	8	48	yes	yes	yes	47	8.3	2

## Data Availability

The data presented in this study are available on request from the corresponding author.
